# Morphological Characteristics of the Sperm of the Peregrine Falcon (*Falco peregrinus*) during the Reproductive Season

**DOI:** 10.3390/vetsci8090169

**Published:** 2021-08-24

**Authors:** Andrea Oliveira, Felisa Martínez, Lydia Gil, Victoria Luño

**Affiliations:** 1 Department of Animal Pathology, Faculty of Veterinary Medicine, Universidad de Zaragoza, 50013 Zaragoza, Spain; chasethedark94@gmail.com (A.O.); felimtz@unizar.es (F.M.); lydiagil@unizar.es (L.G.); 2 Instituto Universitario de Investigación Mixto Agroalimentario de Aragón (IA2), Universidad de Zaragoza, 50013 Zaragoza, Spain

**Keywords:** peregrine falcon, sperm morphology, sperm defects, reproductive season

## Abstract

The morphological characteristics of different sperm cells (normal, abnormal, and immature) in the peregrine falcon during the reproductive season were analysed. We also classified the main sperm defects found in semen. Semen samples were collected from mature peregrine falcons via cloacal massage and stained with Diff-Quik stain. The percentages of normal, abnormal, and immature sperm cells were determined by bright-field optical microscopy. The number of normal spermatozoa were greater at the initial stage and subsequently decreased during the middle and later stages of the reproductive season (*p* < 0.01). In contrast, the percentage of abnormal spermatozoa increased significantly in the middle and end stages of the reproductive season (*p* < 0.05), whereas the proportion of immature spermatozoa remained stable during the study. Head defects represented the greatest proportion of morphological abnormalities, followed by the defects in the tail and midpiece regions. A small percentage of multiple defects and cytoplasmic droplets were also observed in the falcon spermatozoa. The findings of this study might be important for the development of future conservation protocols for falcon sperm.

## 1. Introduction

The peregrine falcon (*Falco peregrinus*) is a cosmopolitan species distributed globally, except in the Pacific Islands and Antarctica [1]. The largest European population of this raptor, especially the subspecies *Falco peregrinus* brookei, is found in Spain. In recent years, several studies have focused on the quantification and evolution of the peregrine falcon population; however, little information is available about falcons in captive breeding programs [2].

The falcon displays some special reproductive features compared to other avian species. It is a monogamous and seasonal bird whose reproductive cycle extends from February to May in Spain [3,4]. The beginning of its breeding season is mainly influenced by the photoperiod, according to the gradual increase in daylight hours [5]. In addition, high temperature peaks can negatively affect semen production and the laying of eggs [6] as well as the natural reproductive behaviour and copula of these falcons [7]. Different strategies are being utilised in falcon conservation programs, such as captive breeding and the creation of bio banking [8,9]. Frozen semen becomes an increasingly relevant tool because it allows the storage of genetic material from several males for a long period of time, even after bird death [9,10]. Avian sperm cryopreservation has some limitations related to the filiform head shape, which makes them more susceptible to injury, and the sensibility in the osmolality changes during freezing–thawing [10]. In recent years, several studies have optimised cryopreservation protocols and the type and concentration of cryoprotectant utilised [11,12,13].

Artificial insemination (AI) in combination with frozen semen are necessary tools for increasing the reproductive success of falcons in captivity, due to the fact that raptors have a single reduced reproductive season each year, where the influence of external environmental factors and the difficulty in finding a compatible mate may negatively impact their chances of reproductive success [2,14]. Therefore, knowledge of the reproductive biological and physiological characteristics of wild birds’ spermatozoa is essential for developing AI and, consequently, for obtaining live raptor chickens [5].

In different species, sperm morphology has been correlated with fertilising capacity [15] and sperm speed, which play an essential role in determining sperm competence and reproductive success [16]. Sperm competition is defined as the evolutionary process that occurs when the spermatozoa of two or more different males try to fertilise the same group of oocytes. Monogamous species, such as the peregrine falcon, do not have this selection pressure; thus, the semen samples tend to present a high degree of pleomorphism, with percentages ranging between 55 and 65 for the immature and abnormal spermatozoa [2,12,17].

Sperm characteristics vary between different peregrine falcon subspecies, individuals, and even between semen samples obtained from the same male due to the limited reproductive season and the difference in the methods used for semen collection [18]. Semen quality parameters such as total volume range from 2 to 222 µL, and concentration from 12.3 to 55.3 × 10^6^ sperm/mL [2,18,19]. In addition, no consensus classification model for sperm defects is available for the peregrine falcon and there are no studies that focus on the types and evolution of sperm abnormalities in the falcon semen. The aim of this study was to determine the morphological characteristics of different sperm cells (normal, abnormal, and immature) in the peregrine falcon during the reproductive season. In addition, we sought to classify the main sperm defects observed in the falcon semen samples.

## 2. Materials and Methods

### 2.1. Reagents and Media

All chemicals were obtained from Merck KGaA (Darmstadt, Germany), unless otherwise indicated.

### 2.2. Animals

The study was approved by the Veterinary Ethical Committee of the University of Zaragoza (PD04/18NE). Animal care and use were carried out in compliance with the ARRIVE guidelines and according to the Spanish Policy for Animal Protection (RD53/2013), which meets the European Union Directive (2010/63/EU) on the protection of animals used for experimental and other scientific purposes. Five mature and healthy peregrine falcons (property of breeding centre Et Reverâ, San Martín de la Vega, Spain) were utilised during the breeding season (February–May). The birds were housed individually in open facilities with a natural photoperiod. They were fed once day, mainly with quails, provided vitamins as obtained from the Masalles Top Complex Peregrinus^®^ (Masalles Europe S.L., Barcelona, Spain), and were granted ad libitum access to water.

### 2.3. Semen Collection and Analysis

Semen was collected using forced abdominal massage [5]. Three semen samples were collected from each of the 5 peregrine falcons (15 sperm samples) in graduated microcapillary tubes with care to avoid contamination with cloacal products. The colour and volume were directly evaluated. The semen samples were classified according to the date of collection: initial (from 15 February to 15 March), middle (from 16 March to 15 April), and later (from 16 April to 15 May) stages of the reproductive season.

Immediately after collection, the sperm motility was subjectively assessed using a light microscope (Leica^®^ DM2500 LED, l’Hospitalet del Llobregat, Spain) with a heated stage at 37 °C and the sperm quality was scored on a scale ranging from 0 (lowest) to 5 (highest). All the samples analysed had a subjective motility higher than 3, and a total volume of 15.8 ± 3.2 µL, to assure the sperm quality. For the sperm morphology assessment, sperm smears were prepared by adding a 5 µL drop of semen to a microscope slide and air dried. Then, samples were fixed in methanol for 1 min and then stained for 1 min in each of the red and blue solutions of the Diff-Quik stain (Everest, Barcelona, Spain). Finally, the smears were rinsed with distilled water and air dried. The percentages of normal, abnormal, and immature sperm cells in each stained sample were determined in 100 randomly selected cells using a light microscope (Leica^®^ DM2500 LED, l´Hospitalet del Llobregat, Spain).

### 2.4. Statistical Analysis

Statistical analysis was performed using IMB SPSS version 22.0 for Windows by IBM Corp. (Chicago, IL, USA). Data concerning normal, abnormal, and immature spermatozoa were analysed using the Chi-squared test. Differences were considered significant if *p* < 0.05.

## 3. Results

### 3.1. Description of the Sperm Subpopulations

**Figure 1:**
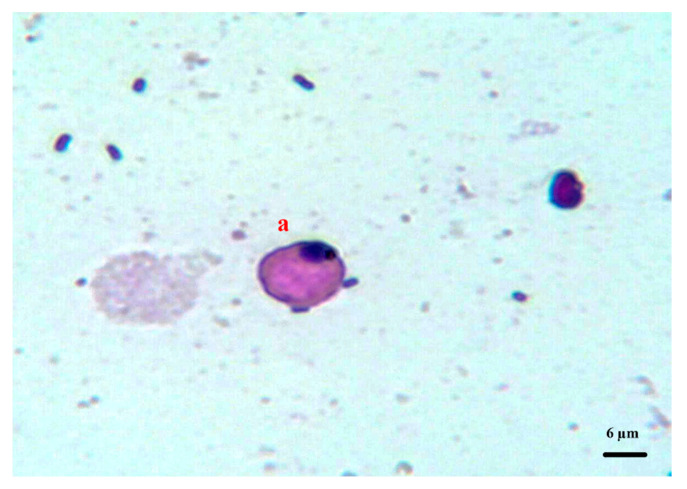
Immature sperm cell (a) stained with Diff-Quik from peregrine falcon semen samples (×1000).

Three different sperm subpopulations were found in all the analysed semen samples: normal, immature, and abnormal spermatozoa. The immature cells were further classified according to their morphological characteristics as follows: round cells, elongated cells with incipient flagellum, or filiform cells with flagellum and a residual body attached to the head (Figure 1).

**Figure 2:**
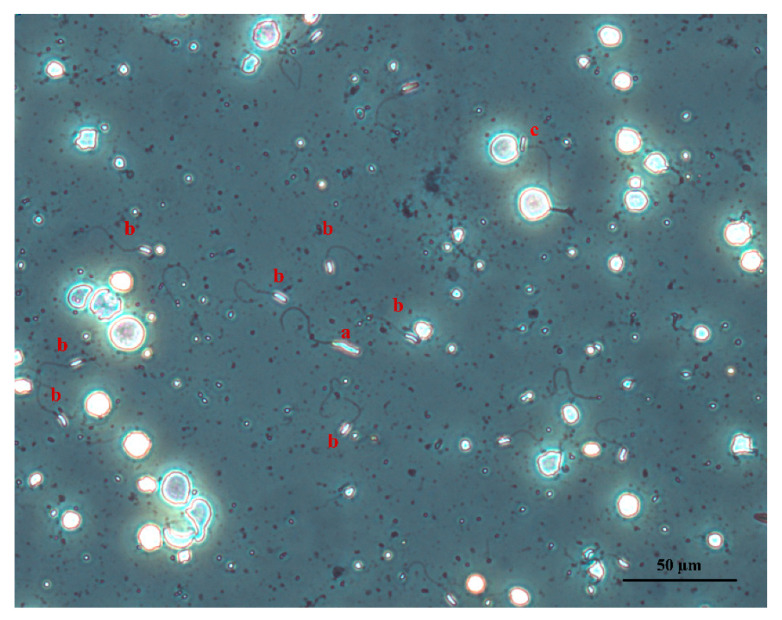
Sperm defects determined in peregrine falcon semen samples. Macrocephalic sperm (a), normal sperm (b), midpiece defect (c) (×20).

Morphological defects were divided into five broad categories: head, tail, midpiece, multiple, and cytoplasmic droplets (Figure 2). Head anomalies included macrocephaly, microcephaly, acephaly, and bent and deformed heads. Midpiece anomalies included a detached head, bent neck, bent midpiece, and coiled midpiece. Tail anomalies included a bent tail, coiled tail, bent tail hook, multiple tails, and a split tail.

**Figure 3:**
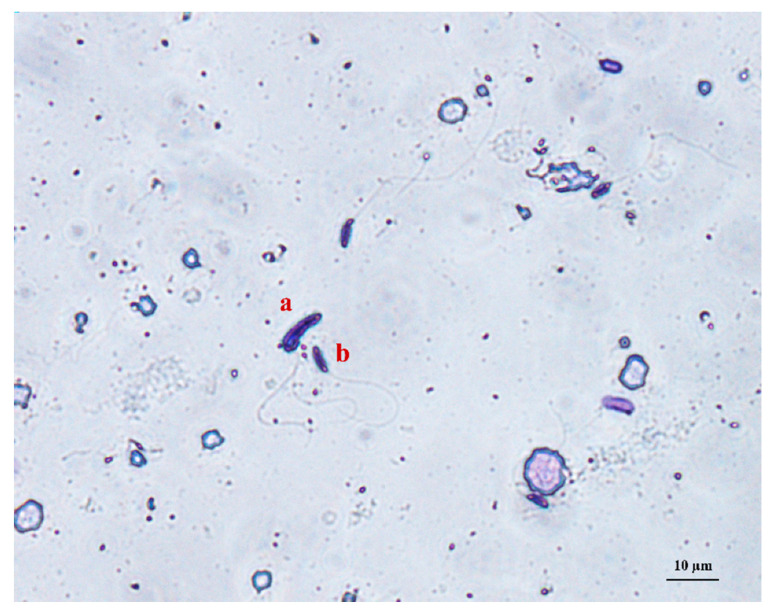
Spermatozoa with multiple defects (macrocephalic head and multiple tails) (a) and normal spermatozoa (b) stained with Diff-Quik from peregrine falcon semen samples (×40).

Multiple defects referred to sperm that displayed a combination of two or more of the above defects (bent heads with multiple or coiled tails, macrocephalic heads with droplets or multiple tails) (Figure 3).

### 3.2. Changes in the Sperm Subpopulations during the Breeding Season

**Figure 4:**
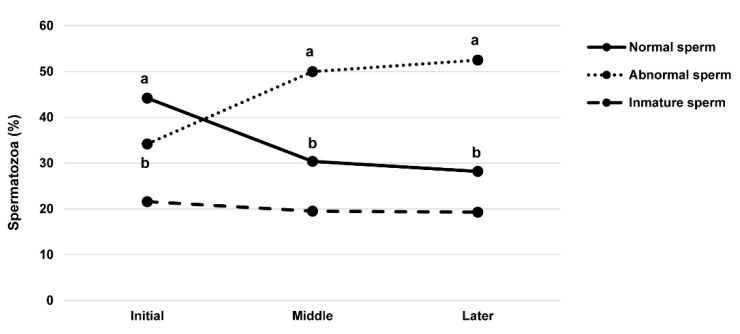
Percentages of the normal, immature, and abnormal sperm in falcon semen samples evaluated in the initial, middle, and later stages of the reproductive season (*n* = 3258). a,b: Different letters indicate significant differences (*p* < 0.05).

The total incidence of the morphologically normal sperm was 34.40%, the immature sperm was 20.86%, and the abnormal sperm was 45.70%, independent of the time of the mating season. No significant differences were found in the proportion of maturity of sperm cells between the different males utilised. The percentages of normal and abnormal sperm subpopulations showed significant differences throughout the reproductive season (*p* < 0.01) (Figure 4). The percentages of normal spermatozoa were greater at the initial stage and subsequently decreased during the middle and later stages of the reproductive season (*p* < 0.01). In contrast, the number of abnormal spermatozoa increased significantly in the middle and end stages of the mating season (*p* < 0.05). The proportion of immature forms remained stable during the study, although it tended to decrease at the end of the breeding season.

The incidence of morphological abnormalities in the falcon semen samples is shown in Table 1. These defects were divided into five categories: head defects (29.2%), midpiece defects (5.36%), tail defects (5.96%), cytoplasmic droplets (2.93%), and multiple defects (1.30%). Head defects represented the greatest proportion of the morphological abnormalities, followed by defects in the tail and midpiece regions. A small percentage of multiple defects and cytoplasmic droplets were also observed in the falcon spermatozoa. The proportion of head defects increased as the reproductive season progressed and the greatest values were observed at the end stage of this study. However, the percentage of tail defects was higher in the later stage than the initial and middle stages of the reproductive season. The other sperm defects did not show differences in the three stages evaluated during the breeding season.

## 4. Discussion

Raptor semen shows special features, such as a high degree of pleomorphism and filiform head shape. In monogamous species, including falcons, the absence of sperm competition causes very heterogeneous sperm populations, with a high proportion of immature and abnormal sperms [17]. The ejaculates of golden eagles (Aquila chrysaetos) have very heterogeneous spermatozoa (>37%) in contrast to polygamous birds, such as chickens, with lower values of abnormal sperm (<20%) [20,21]. In the peregrine falcon, [12] determined the values of 55–65% of spermatogonia and spermatids in semen samples and [22] reported that the proportions of morphologically abnormal sperms ranged between 37 and 75%. In our study, we found a lower percentage of abnormal forms (44.81%) than those from other studies because we divided the semen samples into three different sperm subpopulations: normal, immature, and abnormal forms. In mammals, abnormal sperm values greater than 25% are taken as the semen exclusion criteria due to a decreased fertile potential [23]. In assisted reproductive technologies, the assessment of morphological characteristics is a reliable indicator for predicting the sperm fertilising capacity; therefore, it is important to distinguish between the three sperm subpopulations due to the possible implications related to fertility.

Various staining protocols have been used for semen analysis in birds. Different factors affect the quality of staining, such as the content of ionisable salts, osmotic pressure, pH, concentration, and time of staining and drying, which limits comparability between different studies [24]. Romanowsky-type stains, such as Diff-Quik^®^ stain (Everest, Barcelona, Spain), are useful in revealing structural detail during morphology sperm studies in falcons [2,12,14]. In addition, conventional live/dead stains, such as eosin blue, eosin–nigrosine, eosin blue–aniline, and bromophenol blue–nigrosin could be used in the analysis of sperm morphological characteristics. The authors of [19] investigated eight different supravital staining protocols and their usability to assess vitality in avian spermatozoa. Eosin blue appeared as the most usable live/dead stain in falcons. In a recent study, the conventional stain eosin blue 2% enabled an efficient viability assessment, as well as SYBR^®^ 14/Green–propidium iodide (Merck KGaA, Darmstadt, Germany), showing significant and strong correlations with sperm motility [25].

In this study, we hypothesised that peregrine falcons were in a similar reproductive stage because no difference was found in the proportion of maturity of sperm, and the birds were fed and housed in the same conditions during the research. As the peregrine falcon is a seasonal breeding species, sperm production will be conditioned by the influence of environmental factors, which change during the reproductive season [5]. We determined that modifications in the percentages of the different sperm subpopulations are expected as the mating season progresses. The authors of [26,27] found an increase in the number of abnormal spermatozoa early in the breeding season in the American kestrel (Falco sparvenius) and Sandhill crane (Grus canadensis). However, no differences in sperm motility, normal sperm morphology, or viability were observed between the first and mid-season ejaculates of Pinktail duck [28]. In the present study, the proportion of abnormal sperm was initially low, but later increased in the middle and late stages of the reproductive season until the values became higher than 50%. In falcons, the sperm concentration increases early during the breeding season, peaks in mid-season, and declines thereafter, when testosterone levels are lower than normal [2]. However, only a critical level of circulating testosterone is necessary to produce an adequate percentage of morphologically normal spermatozoa [28]. The reduction in testosterone concentration at the end of the mating season accompanies testes regression and subsequently semen production [29]. Immature sperm remained stable in this study, although they tended to decrease during the reproductive season. These findings may be due to the continuous cell renewal as a consequence of the stimulation produced by daily semen collection. Some authors have suggested that immature sperm function as “push” cells, aiding in the movement of the other sperms through the seminal tubules or from the reproductive tract of the female to the site of fertilisation [30], or as “kamikaze sperm” [31]. Immature sperm, along with leukocytes, have been shown to be the main factors involved in sperm oxidation [32], reducing the sperm function and conservation. However, physiological levels of the reactive oxygen species are essential for sperm capacitation and transduction signalling during fertilisation in mammals [33]. The role of these immature cells remains unknown.

Although several authors have described the normal falcon sperm morphology [25,34], limited information is available on the percentages and types of sperm defects observed in raptors. In addition, no uniform system for classifying the sperm abnormalities has been proposed in avian species, which makes it difficult to perform comparisons. In the current study, falcon semen samples showed relatively higher average values of abnormal sperm (44.5%) than those reported in previous studies. The authors of [22] determined that the percentage of abnormal sperm in the falcon semen ranged from 35.7 to 75.1%, while [2] observed abnormal sperm percentages below 20.0% in several breeds of falcons. In relation to the incidence of specific sperm defects, we found that the abnormal head represented the greatest proportion of morphological abnormalities, followed by defects in the tail and midpiece regions. These results are consistent with those reported in the studies on golden eagle, which indicated that the most common sperm defects are abnormal heads and coiled tails [21]. In ostrich, tail defects constitute the main anomaly, while head defects show comparatively low frequencies [35]. In contrast, head abnormalities formed the greatest proportion of defects in emu [36], similar to the findings of our research with peregrine falcon. Acrosome and mid-piece defects are the most frequent morphological abnormalities observed in turkey and cock semen [37,38]. Cytoplasmic droplets, a sperm defect very common in mammals, exhibit low percentages in avian semen [35]. In quail, as in other birds with long head sperm, the excess spermatid cytoplasm accumulates just behind the acrosome and is lost during spermiation. Consequently, no cytoplasmic droplets remain on the spermatozoon [39].

Sperm head defects are associated with testicular degeneration in mammals [40]. In birds with seasonal reproduction, the testes increase in size around 300–400 times at the beginning of the breeding season and then reduce and lose vascularisation at the end of the season, until they become completely inactive [29]. In this study, the increase in the percentage of head defects during the breeding season may be the result of testicular tissue degeneration. The proportion of immature sperm was stable during the reproductive season, which suggests that as the testicular tissue degenerates, there may be an increase in the production rate of the useful tissues in a compensatory manner [41], resulting in an increase in erratic cell production and abnormal sperm.

A small percentage of sperm showed more than one abnormality simultaneously. Multiple defects included bent heads with multiple or coiled tails, macrocephalic heads with droplets, or multiple tails. Spermatozoa with supernumerary flagella seem to be common in avian species [36,42,43,44], including the peregrine falcon [18]. This type of sperm defect is considered to be a rare defect in mammals that negatively affects sperm motility and fertilising capacity [45]. On the other hand, spermatozoa with one flagellum appear to be produced in greater quantities and at lower energy costs. As a result, semen samples with high proportions of multiflagellate sperm prove to be a disadvantage to the species [46]. Biflagellate sperm are considered to be normal cells in the ejaculates of some teleost fishes [47] and hermaphrodite molluscs [48], with DNA content equivalent to that of somatic cells [49]. Therefore, the presence of macrocephalic biflagellate spermatozoa with duplicated genetic material could be a favourable characteristic for enhanced fertility in birds, although not specifically for fertilisation.

It is still not clear whether high proportions of immature and abnormal sperm play any role in the semen samples of monogamous species. It should be noted that in birds, the eggs are fertilised by a single sperm but depend on polyspermia for the normal development of the embryo in its early stages [50].

## 5. Conclusions

In conclusion, we determined the modification of normal, abnormal, and immature falcon sperm along the breeding season. Head defects represented the greatest proportion of morphological abnormalities, followed by defects in the tail and midpiece sperm regions. The results of this study suggest that the moment of the reproductive season can affect semen sample quality in the falcon.

## Figures and Tables

**Table 1:** Classification and incidence of falcon sperm defects determined at the initial, middle, and later stages of the reproductive season; values are expressed as *n* (%); (*n* = 3258).

Sperm Defects							
Stage Season	Normal Sperm	Immature Sperm	Head	Midpiece	Tail	Cytoplasmic Droplets	Multiple
**Initial**	543 ^a^ (44%)	266 (21.5%)	256 ^a^ (20.7%)	58 (4.7%)	69 ^a^ (5.6%)	33 (2.7%)	10 (0.8%)
**Middle**	307 ^b^ (33.4%)	204 (22.3%)	266 ^a^ (28.9%)	56 (6.1%)	42 ^a^ (4.6%)	28 (3%)	16 (1.7%)
**Later**	285 ^b^ (25.8%)	207 (18.8%)	420 ^b^ (38%)	58 (5.3%)	85 ^b^ (7.7%)	34 (3.1%)	15 (1.4%)

^a,b^ Different letters in the column indicate significant from each other (*p* < 0.05).

## Data Availability

The data that support the funding of this study are available from the corresponding author upon reasonable request.
